# Objective evaluation of similarity scores derived by Evofinder^®^ system for marks on bullets fired from Chinese Norinco QSZ-92 pistols

**DOI:** 10.1080/20961790.2019.1642984

**Published:** 2019-09-09

**Authors:** Feng Dong, Yabin Zhao, Yaping Luo, Weifang Zhang, Yuesong Li

**Affiliations:** a School of Forensic Science, People’s Public Security University of China, Beijing, China; b Shanghai Key Laboratory of Crime Scene Evidence, Shanghai Research Institute of Criminal Science and Technology, Shanghai, China; c Graduate School, People’s Public, Security University of China, Beijing, China

**Keywords:** Forensic sciences, firearm identification, Evofinder^®^, likelihood ratio, automatic correlation

## Abstract

In recent years, many studies have been conducted in the field of firearm identification with the objective of providing an objective method of evaluating the comparison of cartridge cases. However, less attention has been paid to the objective evaluation of bullet comparisons. In this study, 1 000 registered Chinese Norinco QSZ-92 pistols were used, and a database of 2 996 bullets was constructed. Both the receiver operating characteristic (ROC) curve and the score-based likelihood ratio method were used to objectively evaluate the similarity scores derived by the Evofinder^®^ system. The results indicate that this system has excellent ability to discriminate between the selected pistols. This paper proposes an objective evaluation method, which serves as a response to the ongoing debates regarding the foundation of the discipline.

## Introduction

Traditionally, firearm examiners mainly perform identification by carrying out microscopic comparisons, which are labour-extensive and time-consuming. Because image processing techniques have significantly evolved, various automatic ballistic identification systems can satisfy the accuracy requirement when a suspect cartridge case is correlated with a large database.

The Evofinder^®^ system is a second generation electronic comparison system developed by the ScannBI technology company [1]. The performance of the Evofinder^®^ system has been reported by several studies. Rahm [2] proposed a quantitative effectiveness criterion to determine the correlation quality of different comparison systems, and the results demonstrated the good performance of the Evofinder^®^ system. In 2018, Li [3] collected test-fired bullets and cartridge cases fired from registered firearms to construct the registered ballistic database (RBD), and investigated the performance of RBD based on the Evofinder^®^ system. The results revealed that the implementation of RBD is both effective and efficient [3].

Evofinder^®^ uses effective mathematical algorithms to compare the digital signatures and profiles, and ranks them according to the degree of similarity scores. Although the algorithms in the Evofinder^®^ system act as a blackbox for firearm examiners, the main advantages of this system are twofold. On one hand, regardless of choosing from specimen A or specimen B to start a correlation, the similarity score between A and B is consistent, which means that the system is objective and stable. On the other hand, if the RBD has a sister bullet fired from the same firearm with the questioned bullet, the candidate list provided by the system typically includes the sister bullet, which means that the system is reliable.

In practice, the standards for using the similarity scores may vary for different laboratories. Some laboratories use the ranking positions, while others search for gaps between two consecutive scores in the correlation list. The key is to more properly and objectively use the similarity scores. Riva and Champod [4] proposed the use of a score-based likelihood ratio (SLR) method to objectively evaluate the similarity scores of cartridge case marks, and the results revealed that it is feasible to develop an automatic system that can achieve the objective and reproducible evaluation of evidence arising from the similarities/differences between the impressed marks found on the fired primer cups. In 2017, Riva et al. [5] investigated the subclass characteristics on the breech face marks based on the SLR method. To date, studies on evaluating the similarity scores of bullet marks using the likelihood ratio approach have not been reported.

To objectively evaluate the similarity scores of bullet marks, 2 996 bullets fired from 1 000 registered Chinese Norinco QSZ-92 pistols were collected and entered into the Evofinder^®^ system. The receiver operating characteristic (ROC) curve discussed in Section “Evaluation of similarity scores using ROC curve” and the SLR method discussed in Section “Evaluation of similarity scores using SLR” were used to evaluate the similarity scores.

## Materials and methods

### Input of data and correlation

In most Chinese provinces, three rounds of test fire are needed before a registered pistol is distributed to a police officer. We collected 3 000 bullets fired from 1 000 newly manufactured Norinco QSZ-92 pistols, which were sequentially numbered from “00001” to “01000”. The ammunition used was Model DAP92 9-mm ammunition. Each specimen was named using a combination of the pistol name and shot number; for example, 00001-1 is the 1st bullet fired from pistol 00001 and 01000-3 is the 3rd bullet fired from pistol 01000. Except from the four deformed bullets labelled as 00032-B3, 00205-B3, 00273-B3 and 00275-B3, the other 2 996 bullets were entered into the Evofinder^®^ system.

This study considered three main bullet characteristics: the slippage mark, land engraved area (LEA) and groove engraved area (GEA). The slippage mark is generated during the first introduction of the bullet to the barrel and at the beginning of its rotary acceleration. The Evofinder^®^ system provides a result based on the slippage mark, LEA and GEA, with similarity scores ranging from 0 to 1. This study used version 6.3.0 of the specimen analysis system (SAS) within the Evofinder^®^ system.

To form the known match (KM) scores and known non-match (KNM) scores, every 1st bullet from each pistol was used to start a correlation against the rest of the fired bullets. Then, 1 000 correlation lists were exported and analyzed. Theoretically, considering the four deformed bullets will result in 996 lists consisting of two KM scores and 2 993 KNM scores, while the remaining four lists will consist of one KM score and 2 993 KNM scores. However, Evofinder^®^ missed some correlations when running the SAS. Thus, the effectively performed correlations were less than the theoretical correlations. The numbers of effectively performed correlations were 1 985, 1 996 and 1 995 KM scores for the slippage mark, LEA and GEA, respectively, and 2 982 092, 2 992 720 and 2 991 721 KNM scores for the slippage mark, LEA and GEA, respectively.

### Statistical analysis of correlation results

This study used the RStudio software (RStudio, Inc., version 3.4.4, MA, USA) for statistical processing.

#### ROC curve

The ROC curve is a graphical plot illustrating the diagnostic ability of a binary classifier system. Additionally, it is a plot of the true positive rate versus the false positive rate [6]. The lower left point (0, 0) in a ROC graph represents a classifier that never issues a positive classification, while the upper right point (1, 1) represents a classifier that unconditionally issues a positive classification, and point (0, 1) represents a perfect classifier that never issues a false positive [6]. The area under the ROC curve (AUC) numerically corresponds to the performance of a method with a value of one being the perfect classification system and the *y* = *x* diagonal representing a 50/50 chance of correct classification [6]. The value of AUC, which ranges from 0 to 1, represents the probability of a classification system correctly ranking a randomly selected KM comparison higher than a randomly selected KNM comparison.

#### SLR method

The SLR method has become prevalent in forensic science. A more detailed description of the SLR method can be found in [7].

We formulated two firearms hypotheses at the source level:

- Prosecution hypothesis (typically denoted as H_p_): the questioned bullet and suspect bullet were fired from the same firearm/barrel.
- Defence hypothesis (typically denoted as H_d_): the questioned bullet and suspect bullet were fired from different firearms/barrels.

The SLR is based on the ratio of probabilities under these two opposite hypotheses. Somewhat different from the calculation of the likelihood ratio system, the SLR method uses distances (typically the Euclidean distance) or similarities to derive a score in a pairwise comparison. The SLR method has been applied in various branches of forensic science, including fingerprinting [8], firearms [4] and handwriting analysis [9]. The SLR is expressed as follows:

$$\mathrm{SLR}=\frac{f(\left.S_{p}\right|H_{P},I)}{f(\left.S_{d}\right|H_{d},I)}.$$

where *S_p_* and *S_d_* represent the KM scores and KNM scores, respectively; *I* is the background information and ƒ is a probability density function.

## Results and discussions

### Overall distribution of KM scores and KNM scores

Table 1 summarizes several statistics for the KM and KNM scores of the slippage mark, LEA and GEA, respectively. As can be seen, the standard deviation of KM scores is higher than that of KNM scores for these three marks. This reflects the stability of the Evofinder^®^ system, when the system compares two bullets fired from different firearms.

**Table 1.** Descriptive statistics for KM and KNM similarity scores.

Descriptive statistics	Slippage mark		LEA		GEA	
	KM	KNM	KM	KNM	KM	KNM
Minimum	0.042	0.003	0.183	0.092	0.208	0.124
Average	0.595	0.352	0.589	0.181	0.600	0.252
Maximum	0.942	0.899	0.925	0.945	0.949	0.886
Standard deviation	0.138	0.049	0.089	0.019	0.116	0.034
Kurtosis	2.848	5.259	3.168	6.531	3.072	4.137
Skewness	−0.586	−0.236	−0.063	0.815	−0.318	0.671

LEA: land engraved area; GEA: groove engraved area; KM: known match; KNM: known non-match.

**Figure 1.**
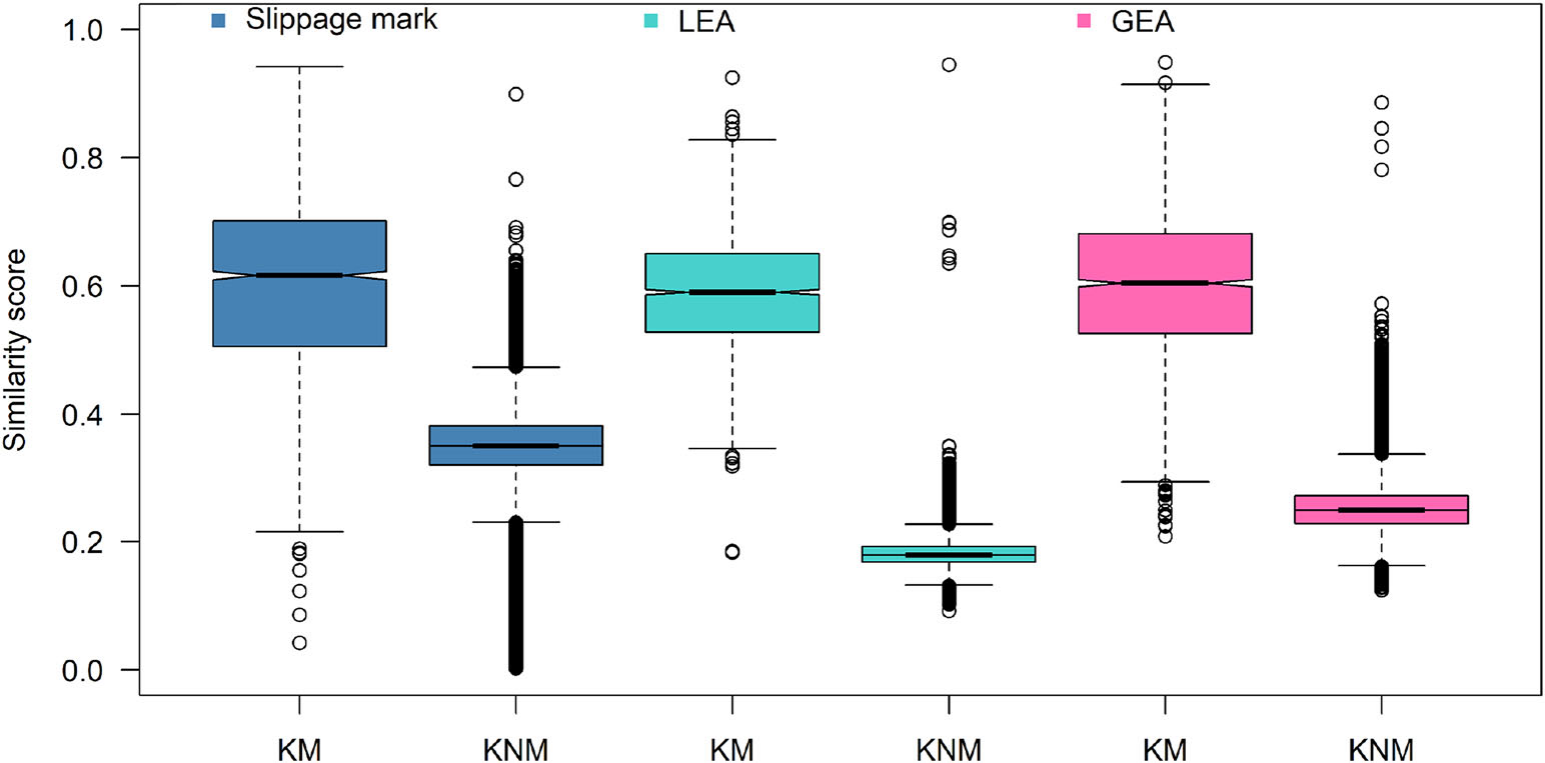
Boxplots of known match (KM) and known non-match (KNM) scores. LEA: land engraved area; GEA: groove engraved area.

Figure 1 shows the boxplot of the KM scores and KNM scores. Approximately all KNM scores are lower than the KM scores, although various outliers exist. The overall KNM score distribution of LEA is lower than that of the KNM scores of the slippage mark and GEA, and the number of outliers for the KNM scores of LEA is lower than that for the slippage mark and GEA.

**Figure 2.**
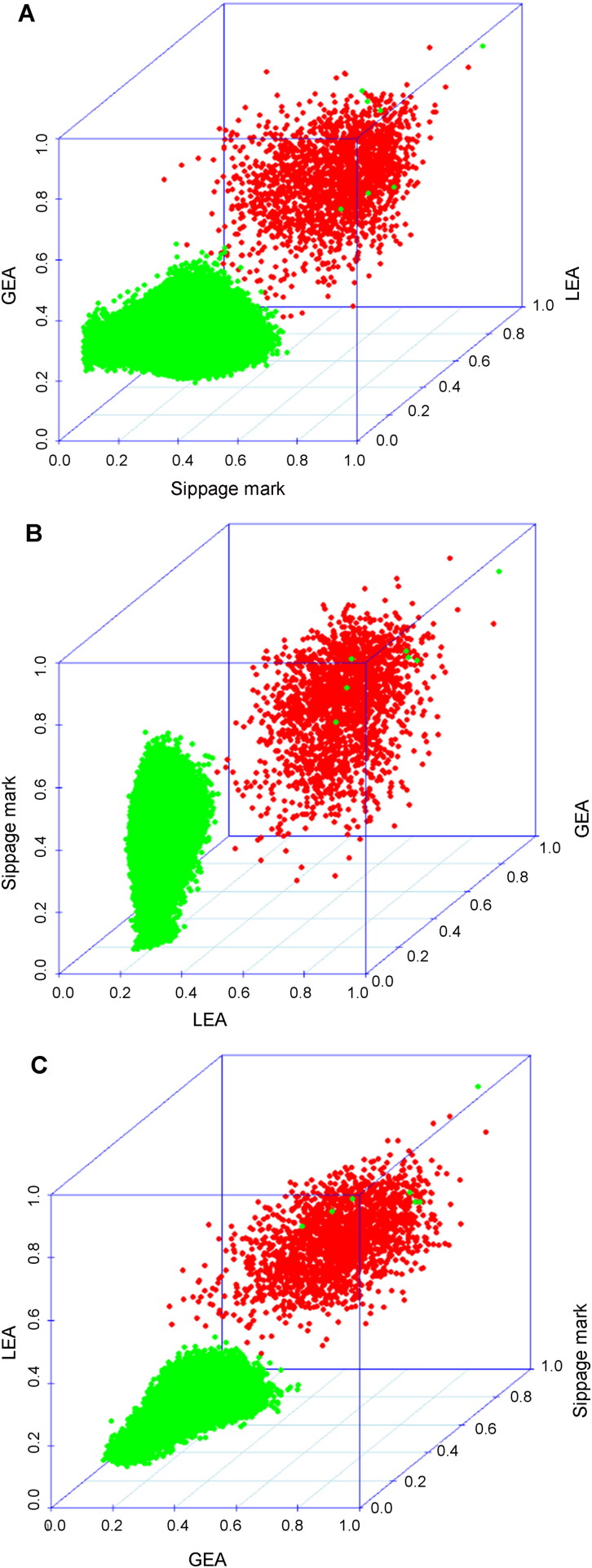
Scatter plots of known match (KM) scores (indicated by red circles) and known non-match (KNM) scores (indicated by green circles) for slippage mark, land engraved area (LEA) and groove engraved area (GEA).

In Figure 2, we combined the scores of three marks for each correlation and plotted the scores as three-dimensional (3D) figures. Notably, we discarded some LEA and GEA scores in the combining process because the SAS within Evofinder^®^ would miss some correlations between the bullets. Additionally, there were only 1985 correlations that had 3D KM scores and 2 982 092 correlations that had 3D KNM scores. Figure 2 shows the conversion of the *x*-axis, *y*-axis and *z*-axis for each figure to view the scores in a more convenient manner. The results revealed a gap between the KM scores and KNM scores. To further verify this point, the supporting vector machine (SVM) method was used to classify the combined scores based on the ground-truth label. The radial basis function was selected as the parameter and the results are presented in Table 2. Although the SVM failed to classify the two KM scores and six KNM scores into the correct results according to the ground-truth label, the accuracy rate was still very high under the abovementioned parameters. Moreover, the specificity and sensitivity were calculated based on the classification result [6]. Specifically, the specificity was approximately 99.99% and the sensitivity was approximately 99.90%.

**Table 2.** Classification results for combined scores based on supporting vector machine (SVM).

	Ground-truth label		
	Known match specimens		Known non-match specimens
Judged as matches	1 983		6
Judged as non-matches	2		2 982 086

### Evaluation of similarity scores using ROC curve

**Figure 3.**
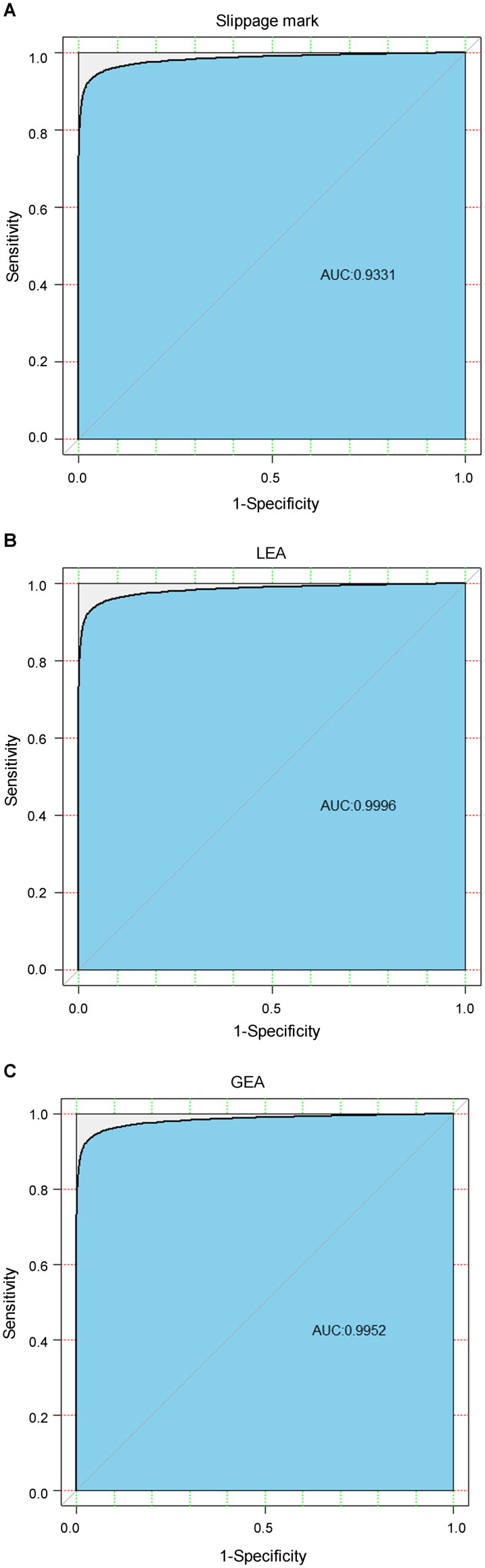
Receiver operating characteristic (ROC) plots of similarity scores for slippage mark, land engraved area (LEA) and groove engraved area (GEA).

Figure 3 shows the ROC plots of the similarity scores for the slippage mark, LEA and GEA, respectively. The AUC values were 0.9331, 0.9996 and 0.9952 for the slippage mark, LEA and GEA, respectively. The results reflect the excellent discriminating power of the Evofinder^®^ system with regard to the marks on bullets fired from Chinese Norinco QSZ-92 pistols.

ROC analysis provides tools for selecting the optimal models and discarding the suboptimal ones irrespectively of (and prior to specifying) the cost context or class distribution. The abovementioned results reveal that the performance of the LEA scores was better than the slippage mark and GEA scores. This may have been caused by the difference between the quality of three mark types, and also by the difference between the relevant algorithms within the Evofinder^®^ system.

### Evaluation of similarity scores using SLR

We carried out the Kolmogorov–Smirnov tests to determine whether the KM score and KNM score distributions were normal, and found that the *P* values were lower than 0.05. Thus, we used the kernel density estimation to fit the distributions of the KM scores and KNM scores.

**Figure 4.**
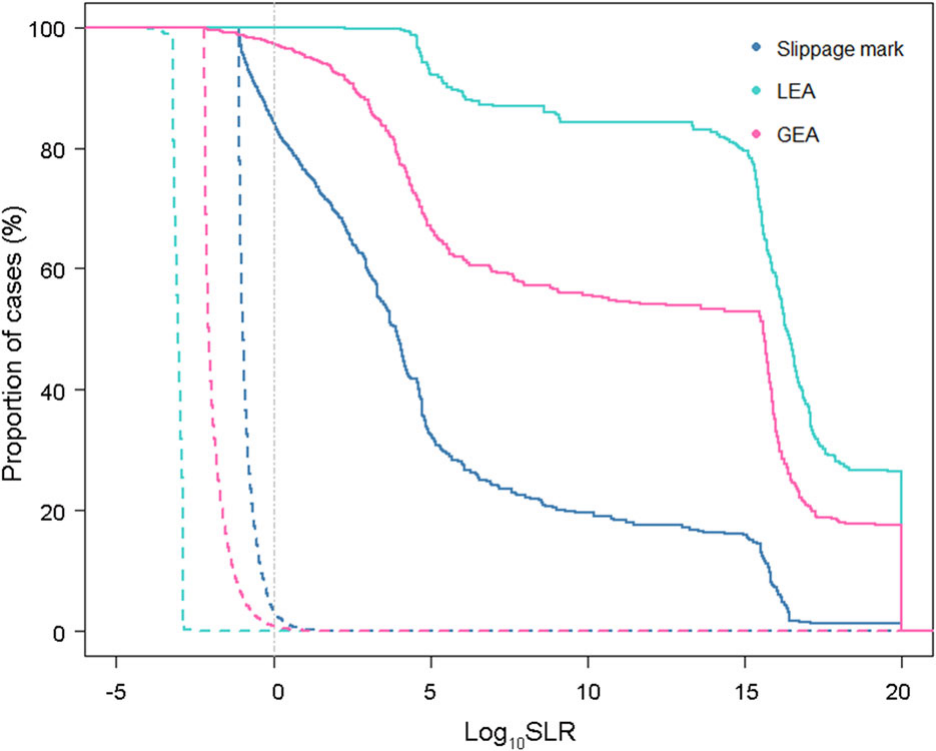
Tippett plot of score-based likelihood ratio (SLR) performance for slippage mark, land engraved area (LEA) and groove engraved area (GEA). The solid and dashed lines indicate true-H_p_ and true-H_d_ curves, respectively.

After the calculation of likelihood ratios, a Tippett plot was used to evaluate the performance of the likelihood ratios. Figure 4 shows the Tippett plot for three bullet marks. A measure for the discriminating power in the Tippett plots is the vertical separation of the true-H_d_ and true-H_p_ curves at a given value on the log_10_ (LR) axis. The more separated the curves were at a given log_10_ (LR) value, the higher was the discriminating power at that value [10]. Figure 4 clearly shows that the LEA had better discriminating power than the slippage mark and GEA.

Table 3 shows the misleading evidence ratio for H_p_ (RMEP) and the misleading evidence ratio for H_d_ (RMED) for three marks. RMEP denotes the false positive rate while RMED denotes the false negative rate. The RMED and RMEP values of the slippage mark were obviously higher than those for the LEA and GEA, which means that the likelihood ratios based on the slippage mark scores were more likely to make Type I and Type II errors.

**Table 3.** Misleading evidence ratio for H_p_ (RMEP) and misleading evidence ratio for H_d_ (RMED) value of slippage mark, land engraved area (LEA) and groove engraved area (GEA).

	Slippage mark (%)	LEA (%)	GEA (%)
RMEP	3.5409	0.0074	0.8740
RMED	15.6171	0.1002	2.7068

The range of SLRs with the corresponding span of scores is summarized in Tables 4 and 5. The SLRs mainly concentrate in the range of 1 000 to infinity for the KM scores of the three marks. According to the verbal equivalents of the calculated likelihood ratio values in Reference [11], the SLRs calculated from the slippage mark, LEA and GEA scores provide very strong support for the same source hypothesis.

**Table 4.** Ranges of scores and correlations of known match (KM) scores given the range of scores for various score-based likelihood ratio (SLR) ranges.

SLR	Slippage mark		LEA		GEA	
	Number of correlations	Scores	Number of correlations	Scores	Number of correlations	Scores
(−∞, 0.1)	138	[0, 0.363)	2	[0, 0.281)	27	[0, 0.309)
[0.1, 1)	175	[0.363, 0.441)	0	[0.281, 0.293)	27	[0.309, 0.353)
[1, 10)	158	[0.441, 0.496)	0	[0.293, 0.305)	40	[0.353, 0.393)
[10, 100)	141	[0.496, 0.541)	1	[0.305, 0.323)	55	[0.393, 0.43)
[100, 1 000)	182	[0.541, 0.583)	3	[0.323, 0.34)	90	[0.43, 0.464)
[1 000, +∞)	1 191	[0.583, 1)	1 990	[0.34, 1)	1 756	[0.464, 1)

LEA: land engraved area; GEA: groove engraved area.

**Table 5.** Ranges of scores and correlations of known non-match (KNM) scores given the range of scores for various score-based likelihood ratio (SLR) ranges.

SLR	Slippage mark		LEA		GEA	
	Number of correlations	Scores	Number of correlations	Scores	Number of correlations	Scores
[10, +∞)	9 870	[0.496, 1)	67	[0.305, 1)	3 908	[0.393, 1)
[1, 10)	95 722	[0.441, 0.496)	154	[0.293, 0.305)	22 240	[0.353, 0.393)
[0.1, 1)	1 077 936	[0.363, 0.441)	430	[0.281, 0.293)	161 275	[0.309, 0.353)
[0.01, 0.1)	1 406 066	[0.302, 0.363)	813	[0.272, 0.281)	1 032 239	[0.257, 0.309)
[0.001, 0.01)	386 845	[0.156, 0.302)	1 457 751	[0.180, 0.272)	1 716 220	[0.191, 0.257)
(-∞, 0.001)	5 653	[0, 0.156)	1 533 505	[0, 0.180)	55 838	[0, 0.140)

LEA: land engraved area; GEA: groove engraved area.

From Table 4, the cut-off points for the slippage mark, LEA and GEA were 0.441, 0.293 and 0.353, respectively. The meaning of the cut-off point is very important. When the similarity score was higher than the cut-off point, the marks on the suspect bullet and questioned bullet were more likely if the bullets were fired from the same firearm. Moreover, when the similarity score was lower than the cut-off point, the marks on the suspect bullet and questioned bullet were more likely if the bullets were fired from different firearms. The calculation of the cut-off points can help firearm examiners make a quick decision with regard to selecting the suspect bullet(s) from the correlation list.

### Limitations

Morrison and Enzinger [12] argues that the SLR method is not scientifically valid. In his opinion, the SLR method only considers the similarity, while the real likelihood ratio should be the similarity divided by the typicality. In this paper, we do not make any remarks regarding Morrison’s argument. If the SLR method actually disregards the typicality, the calculation results will be lower than the actual likelihood ratios, which supports our conclusions.

This study did not use the multivariate model to fit the combined 3D scores of the slippage mark, LEA and GEA. Future work can investigate the added value of using a multivariate model to improve the performance of the SLR method.

## Conclusion

The data presented in this study were used to objectively evaluate the similarity scores of bullet marks derived by the Evofinder^®^ system. The ROC curves and AUC values validated the excellent discriminating power of the Evofinder^®^ system. The results also revealed that, in practice, firearm examiners should use the LEA scores more than the slippage mark and GEA scores to select the suspect bullet(s) from the database.

Moreover, the SLRs were calculated based on the similarity scores. We summarized the range of scores corresponding to the range of SLRs, which allows firearm examiners to use the similarity scores in a more objective and convenient manner.

This study provides firearm examiners with an approach of objective and transparent evaluation when performing identification using an automatic ballistics identification system.
